# Performing central venous catheters in neonates and small infants undergoing cardiac surgery using a wireless transducer for ultrasound guidance: a prospective, observational pilot study

**DOI:** 10.1186/s12887-021-02822-w

**Published:** 2021-08-13

**Authors:** Judith Schiefer, Paul Lichtenegger, Daniel Zimpfer, Doris Hutschala, Lorenz Kuessel, Alessia Felli, Stephan Hornykewycz, Peter Faybik, Eva Base

**Affiliations:** 1 Division of Cardiothoracic and Vascular Anesthesia, Department of Anesthesia, Intensive Care Medicine and Pain Medicine, Medical University of Vienna, Waehringer Guertel 18-20, 1090 Vienna, Austria; 2 Division of Anesthesia and General Intensive Care Medicine, Medical University of Vienna, Department of Anesthesia, Intensive Care Medicine and Pain Medicine, Waehringer Guertel 18-20, 1090 Vienna, Austria; 3 Division of Cardiac Surgery, Department of Surgery, Medical University of Vienna, Waehringer Guertel 18-20, 1090 Vienna, Austria; 4 Department of Obstetrics and Gynecology, Medical University of Vienna, Waehringer Guertel 18-20, 1090 Vienna, Austria

**Keywords:** Wireless ultrasound, Central venous catheter, Ultrasound guided catheter placement, Infants, Congenital heart disease

## Abstract

**Background:**

Neonates and small infants with congenital cardiac disease undergoing cardiac surgery represent major challenges facing paediatric anaesthesia and perioperative medicine. Aims.

We here aimed to investigate the success rates in performing ultrasound (US) guided central venous catheter insertion (CVC) in neonates and small infants undergoing cardiac surgery, and to evaluate the practicability and feasibility of thereby using a novel wireless US transducer (WUST).

**Methods:**

Thirty neonates and small infants with a maximum body weight of 10 kg and need for CVC before cardiac surgery were included in this observational trial and were subdivided into two groups according to their weight: < 5 kg and ≥ 5 kg. Cannulation success, failure rate, essential procedure related time periods, and complications were recorded and the clinical utility of the WUST was assessed by a 5-point Likert scale.

**Results:**

In total, CVC-insertion was successful in 27 (90%) of the patients and the first attempt was successful in 24 (78%) of patients. Success rates of CVC were 80% < 5 kg and 100% ≥5 kg. Comparing the two groups we found a clear trend towards longer needle insertion time in patients weighing < 5 kg (33 [28–69] vs. 24 [15–37]s, *P* = .07), whereas, the total time for catheter insertion and the duration of the whole procedure were similar in both groups (199 [167–228] vs. 178 [138–234] and 720[538–818] vs. 660 [562–833]s. In total, we report 3 (10%) cases of local hematoma as procedure-related complications. Assessments of the WUST revealed very good survey results for all parameters of practicability and handling (all ratings between 4.5 and 5.0).

**Conclusion:**

Although difficulties in CVC-placement seem to relate to vessel size and patient’s weight, US guided CVC-insertion represents a valuable, fast, and safe intervention in neonates and small children undergoing cardiac surgery. Using the WUST is feasible for this clinical application and may aid in efforts aiming to optimize perioperative care.

**Trial registration:**

Wireless US-guided CVC placement in infants; Clinicaltrials.gov: NCT04597021; Date of Registration: 21October, 2020; retrospectively registered.

## Background

Neonates and infants with congenital cardiac disease need to undergo surgical correction or palliative procedures at a given time point, depending on the underlying diagnosis. When undergoing open cardiac surgery, optimized perioperative care is compulsory for these high-risk procedures. A central venous catheter (CVC) is placed as part of routine, in order to provide a magnitude of management goals including drug and volume therapy, as well as the central venous pressure monitoring.

Placing CVCs in children, particularly in neonates and small infants, is far more challenging than in adult patients, which, besides the relative position to the carotid artery and the neck length, partly appears to depend on vein size [1]. Further, vein size is known to correlate with weight, size and age [2]. In addition, central venous cannulation in paediatric patients with congenital heart disease may turn out to be even more difficult due to frequent previous catheterizations, common aberrant vessel anomalies, pre-existent thrombotic occlusion of vessels and preoperative anticoagulant medication, influencing morbidity and mortality [3].

In order to optimize perioperative care by attenuating or preventing possible complications related to CVC’s, critical re-evaluation of practice patterns in CVC-placement in children is demanded. High first attempt rates, low procedural times and low complication rates could contribute to a better outcome [4, 5] .

The use of US guidance for CVC-placement has evolved standard of care in many centres [6, 7]. Using US guidance is known to be superior to the surface anatomy landmark technique for CVC-placement in children [8–11]; reduction of time and number of attempts, as well as the reduced incidence of accidental puncture of the carotid artery with subsequent hematoma formation are important goals, which are achieved by the additional use of US guidance [12, 13]. A potential problem in the use of conventional US is the probe-connecting wire, which sometimes hinders the fluency and handling during the procedure and might inhere the risk of cross- infections. Recently, a novel wireless, bluetooth US transducer has been released and might simplify the use of US in cannulation. US guided axillary vein cannulation using a wireless US transducer (WUST) was described as a feasible, fast, and safe method for the implantation of cardiovascular implantable electric devices [14].

The aim of this study was to evaluate (i) the weight-dependent success rates in performing US guided CVC in neonates and infants undergoing cardiac surgery and to (ii) evaluate the practicability of applying a WUST for CVC-placement in these patients.

## Methods

### Patients

In this prospective single-centre observational pilot study, neonates and small infants with a maximum body weight of 10 kg and need for insertion of a CVC before cardiac surgery at our institution were included between August 2018 and January 2019 and adhered to the applicable Consolidated Standards of Reporting Trials (CONSORT) guidelines. The patient cohort was subdivided into two groups according to their weight: < 5 kg and ≥ 5 kg body weight. Patients with a persistent left superior caval vein or who had undergone previous cardiac surgery or any cardiac intervention before were also included. There was no further inclusion- or exclusion criteria. The study was approved by the local institutional ethics committee of the Medical University of Vienna (institutional review board Nr.1386/2018) and all parents gave written informed consent and agreed to data collection. The study has been performed according to the Declaration of Helsinki. Data curation was performed by using a predefined standardized form. All data was recorded peri-procedurally by an anaesthesiologist who was not involved in the procedure.

Patient characteristics included baseline characteristics, diagnosis, medical history, preoperative medication, and laboratory values before and up to 7 days after the surgical procedure. Increased bleeding risk was assessed by the preoperative coagulation profile, and by the intake of coagulation-modifying medication. Besides, a review of previous hospital reports, echocardiographic, and cardiac catheterization studies, if available, was performed in order to evaluate the vascular anatomy.

### Details of CVC-placement

Five specialists in anaesthesiology, well-trained in paediatric anaesthesia care performed CVC-placement according to local standards. All anaesthesiologists performed between 4 and 7 cases, were adequately trained and experienced with the conventional use of real-time US for performing CVC-placement. In all patients Trendelenburg position (30°) was applied [15] and the CVC was placed into the internal jugular vein using the Seldinger technique under general anaesthesia, appropriate ventilator settings with application of 5 cm H2 positive end-expiratory pressure, and under hemodynamic stable conditions. The catheter size and length was chosen according to the patients’ height and weight. Vessel localization, venipuncture, and guidewire insertion was performed according to the recommended 6-step approach [5] and confirmed using US guidance. The B-mode (2D) was primarily used to detect and visualize the presence of the internal jugular vein according to its collapsibility; the colour flow Doppler and/or the pulsed wave Doppler have been additionally applied to confirm the diagnosis and to definitely differentiate between the carotid artery and the internal jugular vein.

Details of CVC-placement were recorded on standardized forms and included cannulation success rates, number of attempts, multiple attempts (defined as more than 3 attempts), the cannulation time (skin puncture to successful guidewire insertion), the catheter insertion time (skin puncture until intravenous placement of the catheter before external fixation), the duration of the whole procedure (from sterile preparation to external fixation of the catheter), and the incidence and type of CVC related complications. Successful CVC-placement was defined as catheter placement into the internal jugular vein, regardless the number of attempts; failed cannulation was defined as inability to insert the catheter, with subsequent change of the insertion site, or need for open surgical catheter placement.

### The wireless US transducer

**Fig. 1:**
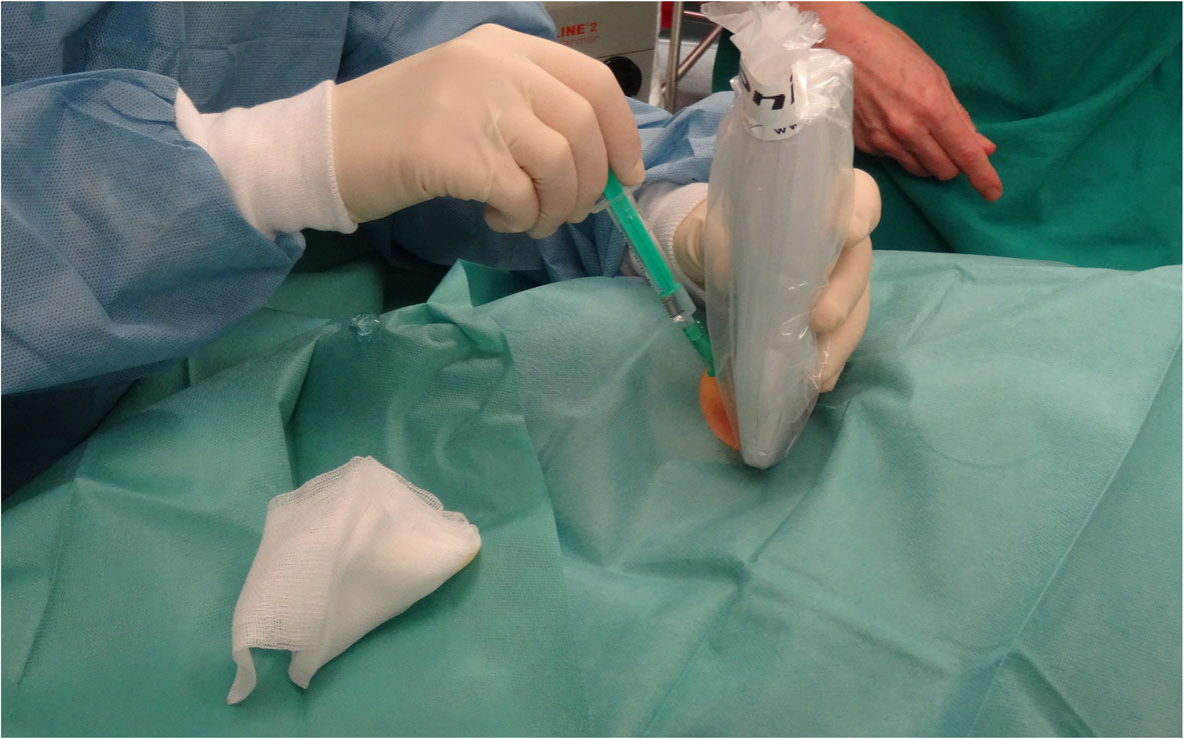
Clinical application of the wireless ultrasound transducer

For US guidance, a blue tooth based WUST (ACUSON Freestyle™Ultrasound, Siemens Medical Solutions, USA, Inc., Mountain View, CA, USA) was applied in all patients after preparation with a proper sterile cover. The device applied in this study consists of an 8- to 3-MHz linear array, communicating with a portable receiving unit including a screen for image visualization. No hockey stick shape linear probe was provided by the manufacturer. The transducer is powered by a removable battery with 90 min endurance. The depth of the image is modifiable within a range from 2 cm to 9 cm. Both, the probe and the receiving unit allow modification and adjustment of the image settings. Fig. 1 shows the clinical application of the wireless ultrasound transducer

In this study, fine adjustment of the image was obtained by the operator itself or an assisting person, according to the operator’s instructions. The practicability of using the WUST for CVC-placement was assessed by a 5-point Likert scale (ranging from 1 to 5; 1 = very poor, 2 = poor, 3 = acceptable, 4 = good, 5 = very good) in questionnaires completed by the CVC placing anaesthesiologist after the procedure. Further, the performing anaesthesiologist had the opportunity to add written comments, firstly to further specify the reason for the rating and, secondly, to give additional feedback.

All anaesthesiologists involved in the study received a standardized introduction for the use of the new US-system.

### Statistical analysis

Statistical analyses were performed using R Studio version 3.6.1 (RStudio, Inc., Boston, MA, USA, https://www.r-project.org/). In descriptive analysis, metric variables were described by mean ± standard deviation or median [range from minimum to maximum], as appropriate. Categorical variables were described by absolute and relative frequencies. The Kolmogorov Smirnov Test was used to assess normal distribution. The Mann–Whitney U tests was used to compare pre-defined endpoints between the two groups in order to investigate independent samples according to their distribution. All *P*-values < .05 were considered as statistically significant.

## Results

In total, 30 neonates and small infants undergoing cardiac surgery were enrolled in this pilot study; seven of them (23%) were neonates at the moment of surgery. Patient characteristics are presented in Table 1.

**Table 1:** Patient characteristics

Characteristics	all patients (***n*** = 30)	< 5 kg (***n*** = 15)	≥5 kg (*n* = 15)	*p*-value
male/female, n (%)	13(43) /17(57)	6(40)/9(60)	7(47)/8(53)	
Age, months, median [IQR]	5 [2–11]	2 [0–3.5]	13 [7–15]	
Weight, kg, mean ± SD	5.80 ± 2.9	3.6 ± 0.6	8 ± 1	
Height, cm, mean ± SD	63 ± 13	54 ± 6	73 ± 6	
Cyanotic, n %	6 (19)	1 (7)	5 (33)	0.16
Antiplatelet therapy, n %	3(10)	0	3(20)	0.2

Data are given as mean ± standard deviation or median [IQR]. Categorical variables are described by absolute and relative frequencies. *Abbreviations: CHD* congenital heart disease, *s* seconds, *SD* standard deviation

The mean weight of all included paediatric patients was 5.8 ± 2.4 kg. As the difficulty of CVC-placement in small children additionally seems to depend on the patients’ weight, we divided our patients into two groups < 5 kg weight (*n* = 15), and patients ≥5 kg weight (*n* = 15). Seven of the included patients were neonates; all neonates belonged to the < 5 kg group. According to the preoperative results of cardiac catheterization, no anatomical anomalies of the vessel course could be revealed by application of the US-guidance for CVC-insertion. Five patients, of whom 4 patients from the ≥5 kg group, had undergone previous heart surgery, and thus, prior catheterization. Three patients weighing ≥5 kg were treated with single- antiplatelet therapy immediately preceding surgery (*p* = 0.2). Table 2. provides data regarding details of CVC-placement.

**Table 2:** CVC details

CVC Details	all patients (***n*** = 30)	< 5 kg (***n*** = 15)	≥5 kg (***n*** = 15)	***p***-value
Overall success, n (%)	27 (90)	12 (80)	15 (100)	0.22
First attempt success, n (%)	24 (78)	11 (73)	13 (86)	0.65
Success within 3 attempts, n (%)	27 (90)	12 (80)	15 (100)	0.22
Number of attempts, median [IQR]	1[1–3]	1 [1–5]	1 [1–2]	0.33
Hematoma, n (%)	3 (10)	3 (20)	0	0.22
Arterial puncture, n (%)	0	0	0	
**Continuous outcomes**				
Needle insertion time, s, median [IQR]	32 [15–42]	33 [28–69]	24 [15–37]	0.07
Catheter insertion time, s median [IQR]	204 [158–230]	199 [167–228]	178 [138–234]	0.67
Duration whole procedure, s, mean ± SD	670 [560–811]	720 [538–818]	660 [562–833]	0.96

Data are given as mean ± SD or median [IQR]. Categorical variables are described by absolute and relative frequencies. *Abbreviations: CHD* congenital heart disease, *CVC* central venous catheter, *s* seconds, *SD* standard deviation

In total, CVC-placement was successful in 27 (90%) patients, the first attempt was successful in 24 (78%) of patients. In paediatric patients weighing < 5 kg the first attempt was successful in 11 out of 15 patients (73%), and in the ≥5 kg group in 13 out of 15 patients (86%). The overall success rate within 3 attempts was 90% (i.e. in 27 patients); in children ≥5 kg the success rate within 3 attempts was 100%, whereas in the smaller weighing group a success rate of 80% was reached within 3 attempts. Multiple attempts, defined as more than 3 needed attempts for successful cannulation, were necessary for 1 patient of < 5 kg. The number of attempts needed for successful cannulation, were slightly, but not significant, higher in the group < 5 kg (1.5 ± 1.1 vs. 1.1 ± 0.4, *p* = 0.33).

**Fig. 2:**
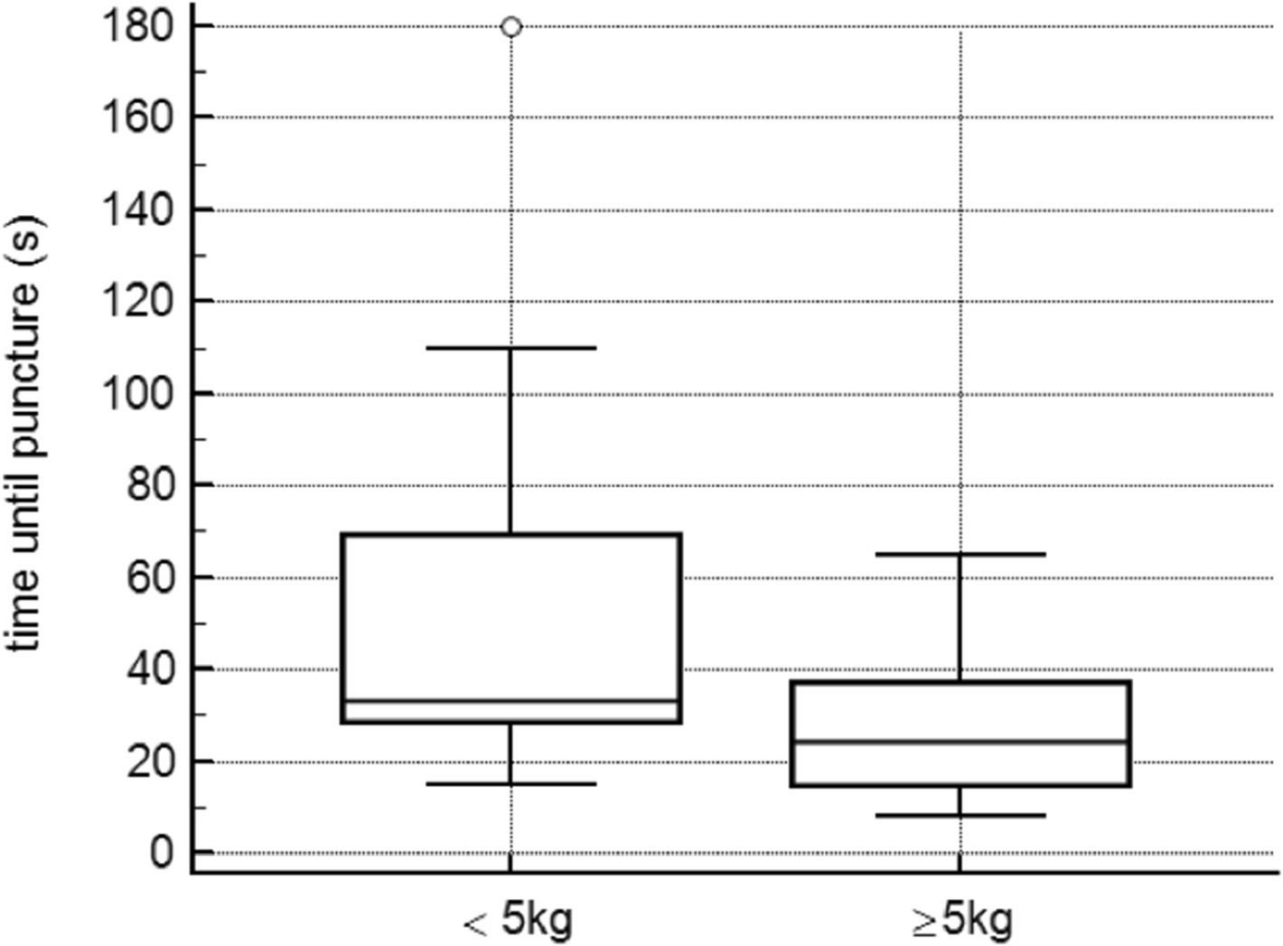
Time until puncture comparing the two groups according to their weight; a clear tendency towards lower needle insertion time is seen in the group ≥5 kg body weight

**Fig. 3:**
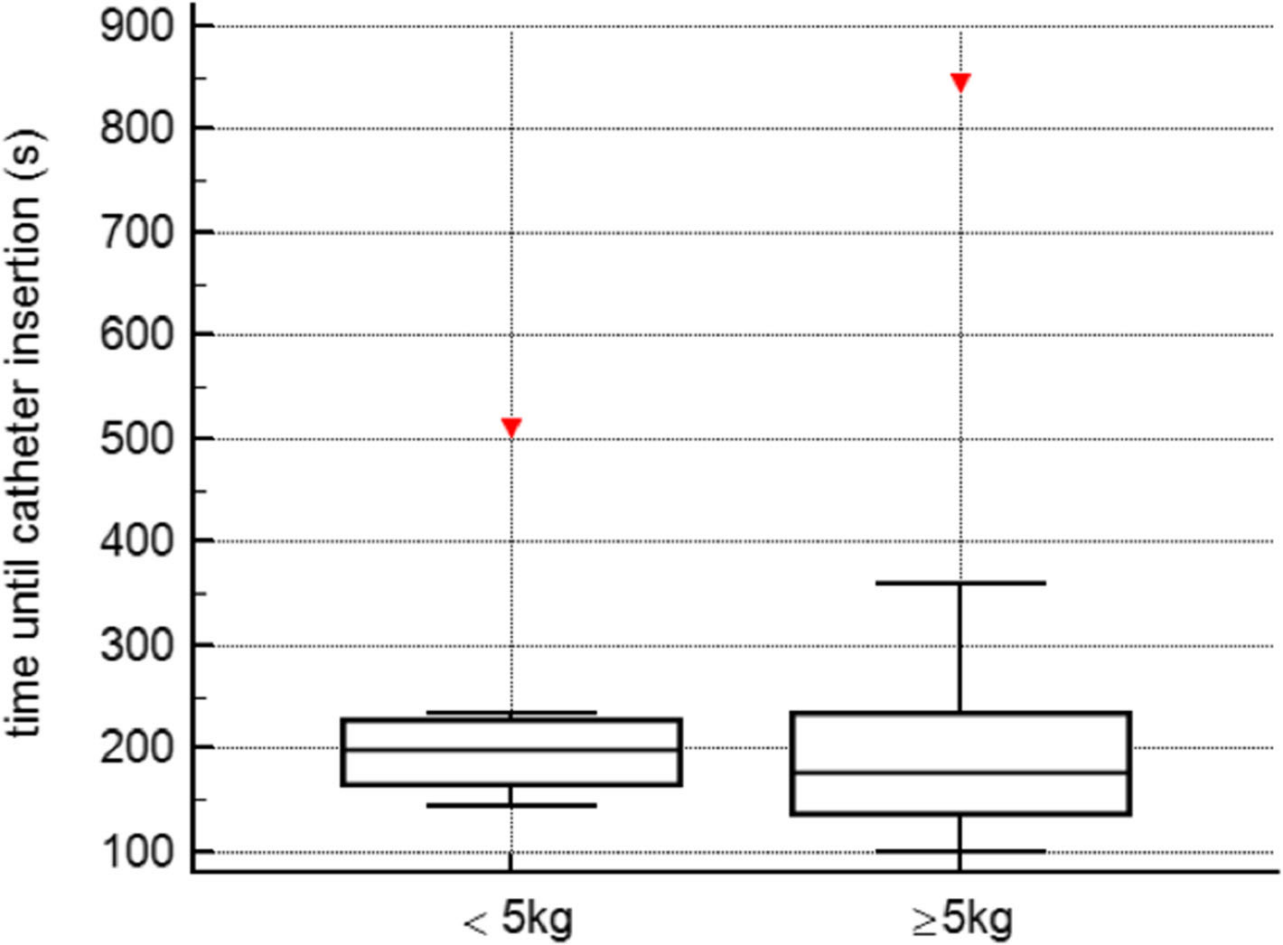
Time until catheter insertion comparing the two groups; the graph shows similar results in both groups

The overall median procedural time in all patients was 670 [560–811]seconds; the median cannulation time (i.e. needle insertion time) was 32 [15–42]seconds, the median duration until successful catheter insertion was 204 [158–230 s. Comparing the two groups we found a clear trend towards lower needle insertion time (i.e. cannulation time) in patients weighing ≥5 kg (24 [15–37] seconds vs. 33 [28–69] seconds, *p* = 0.07), even though not significant. Catheter insertion time and duration of the whole procedure were comparable in both groups. Further details and placement times are depicted in Table 2 and Figs. 2 and 3.

Regarding complications, we report 3 cases of local hematoma due to increasing number of attempts, which subsequently led to failed catheter insertion in all 3 cases; all cases of hematoma formation took place in neonates, weighing < 5 kg; none of those occurred in patients with preoperative intake of antiplatelet medication. Beyond, failed catheter insertion occurred regardless of the performing anaesthesiologist. Successful puncture of the vein, but subsequent failed insertion of the guidewire at the first attempt happened in 6 cases, 4 of whom in the group < 5 kg. Further, no CVC-related infection or thrombosis was observed in our patients during the first postoperative week.

The survey results of the US-practicability are depicted in Table 3.

**Table 3:** US practicability - survey results

The US-probe is easy to handle	4.6 ± 0.6
The visualization of the vessel is good	5 ± 0
Image quality is good	4.9 ± 0.3
The visualization of the needle is good	4.5 ± 0.6

Rating according to Likert scale is depicted as mean ± standard deviation

The practicability and handling of applying the WUST was rated with a mean score of 4.6 ± 0.6, according to the Likert Scale. In all cases the operating anaesthesiologist was able to easily identify the relevant anatomical structures; the visualization of the vessel was rated with very good (5 ± 0 points) by 100% of the operators and even the visualization of the needle was rated with 4.5 ± 0.6. The image quality was scored with 4.9 ± 0.3 points. No workflow interference has been reported in any of the cases. The single-operator use has been rated as a valuable additional option by all users. However, all operators expressed concerns regarding the relatively high weight of the WUST compared to conventional transducers usually applied for paediatric vessel visualization.

## Discussion

Neonates and small infants with congenital cardiac disease undergoing cardiac surgery represent major challenges facing paediatric anaesthesia and perioperative medicine. We here aimed to investigate the success rates in performing US guided CVC in neonates and infants under 10 kg of body weight undergoing cardiac surgery, and to evaluate the practicability of thereby using a WUST.

We found very high total success rates for CVC-placement and first-attempt rates, as well as low median total procedural times, when compared to other studies on this topic [6, 10, 13, 16], and absence of serious complications related to CVC-placement. Although difficulties in CVC-placement seem to relate to vessel size and patient’s weight, we here show that US guided CVC represents a feasible, fast, and safe intervention in neonates and small children undergoing cardiac surgery.

Moreover, we could demonstrate, that the assessment of the applicability of the WUST used in our study revealed high rates on the Likert scale, and that the use of the WUST is feasible for clinical application in both neonates and infants.

Central venous catheters are mandatory for numerous interventions including cardiac surgery in neonates and children. However, CVC-placement can be challenging and multiple anatomic sites may have to be attempted and an unsuitable length of time may be needed to gain access. Many of the paediatric patients undergoing cardiac surgery need multiple surgeries and cardiac catheterization procedures, which makes it important not to use multiple anatomic sites in order to protect the vessels. Moreover, it appears that difficulties in CVC-placement relate to vessel size and are therefore influenced by the body weight of young patients. Therefore, the valuable information- offered by US guidance in general - about the potential anatomical peculiarities is even more important in this delicate patient population. However, application of US guidance for CVC-placement in paediatric patients might especially be challenging due to the small diameter of the vascular structures and deserves scientific attention. In the present study, we report a total success rate for CVC-placement of 90%, a first-attempt rate of 78%, a mean total procedural time of about 11 min, and absence of serious complications related to CVC-placement. When comparing patients < 5 and ≥ 5 kg of body weight, we found that first attempt success rates, success rates within 3 attempts, as well as overall success rates were slightly lower in the group < 5 kg. In addition, we found a clear trend towards higher needle insertion time in patients weighing < 5 kg. We think that this clearly strengthens the hypothesis relating difficulties in CVC-placement to vessel size and patient’s weight.

Since also very small children were included in our study (50% children with a body weight of < 5 kg, the smallest 2.6 kg of weight), we furthermore conclude that our management of US guided CVC-placement is very well applicable and safe in neonates and small children undergoing cardiac surgery. Previous studies investigating CVC-placement in critically ill children under 10 kg body weight reported similarly high success rates, and comparable low complication rates, but longer procedural durations [3, 8, 10, 11, 17]. In contrast to other trials, no inexperienced operator has been assigned with US-guided CVC-placement in our study, which might contribute to the low complication rates and relatively low procedural times.

The most frequent complications of central venous catheterization are arterial puncture or hematoma formation, making further attempts more difficult and leading to high failure rates. Furthermore, the incidence of carotid artery puncture is known to be even higher in children younger than 5 years [18]. In this observational study, the incidence of carotid artery puncture was 0%, which in the author’s opinion is clearly related to the US guidance allowing for the good visualization and clear distinction of the patients’ arteries and veins. Local, subcutaneous hematomas at the puncture site were observed in 3 patients, which can be judged minor critically: hematoma formation occurred in 3 neonates weighing between 2.6 and 3.2 kg (20% of the group < 5 kg) due to an increasing number of attempts, which subsequently led to failed catheter insertion in all 3 cases. The preoperative intake of single- antiplatelet therapy had no influence on peri-procedural hematoma formation.

No serious complications such as pneumothorax, catheter malposition, air embolism, or persistent arrhythmias could be observed; no thrombotic or infectious complications occurred within the first week after insertion, underlining the fact that the risk of bloodstream infections related to CVCs is not significantly increased until the seventh day [3]. Beyond that, long-term complications were not evaluated in our study.

Regarding the applicability of the WUST, effectiveness, time consumption, and safety of the first-in-human experience in axillary vein cannulation guided with this novel WUST for the implantation of cardiovascular implantable electric devices has already been studied and proven recently in adult patients [14]. Further, the application of WUST has recently been shown to be of great value, facilitating procedures in COVID-19 patients [19]. We can state that its use is clinically feasible and suitable for neonates and small infants. The assessment of the applicability of the WUST used in our study revealed high rates on the Likert scale: all participant anaesthesiologists pointed out the very good visualization of the vessel, as well as the very good image quality. In open feed-back comments, application of the WUST has been described as expeditious and user-friendly, since also modifications and adjustments of the image settings could be performed by the operator from the probe’s built-in controls without the assistance of a second operator. The most stated drawback was the higher weight of the transducer, which could increase the likelihood of compressing the internal jugular vein, especially with the decreasing vessel diameter in neonates and small infants, and subsequently, might limit its use in small neonates.

Limitations of our study are the observational design without a comparison group using conventional US transducers, as well as the relatively small number of patients included. Hence, the limited number of patients and subsequently low number of cannulations makes the between-groups statistical testing of the complication rate, as well as the comparison of the procedure-related times, underpowered. Furthermore, comparability regarding complication rates with trials including higher numbers of patients might be limited. However, the homogeneity of our investigated cohort including the range of weight was far higher than in larger trials dealing with this topic [8, 16, 20]. The prospective nature and robust design of our study investigating a well-characterized patient cohort that was monitored closely throughout the entire procedure are strengths of our study.

Given the complexity of the diseases, the procedures, and the related perioperative care, every attempt to attenuate and prevent possible complications is of utmost importance. Therefore, a continuous critical re-evaluation of routinely applied procedures is necessary. Randomized trials on larger study cohorts are needed to further evaluate the potential advantages of the WUST-technology compared to the conventional US-technique.

## Conclusions

US guidance in CVC-placement is of high clinical value offering gain in safety and quality in perioperative management. Although difficulties in CVC-placement seem to relate to vessel size and patient’s weight, US guided CVC represents a valuable, fast, and safe intervention also in neonates and small children undergoing cardiac surgery. Using the WUST is feasible for this clinical application and may aid in efforts aiming to optimize perioperative care.

## Data Availability

Data generated or analysed during this study are mainly included in this published article. The complete dataset used and/or analysed during the current study are available from the corresponding author on reasonable request.

## Abbreviations

CHD: congenital heart disease
CVC: central venous catheter
US: ultrasound
WUST: wireless ultrasound transducer
